# A Novel NPHS1‐Associated Phenotype Characterized by Recurrent Transient Proteinuria

**DOI:** 10.1155/crin/4258555

**Published:** 2026-06-12

**Authors:** Etsuko Tanaka, Takao Konomoto, Hiromi Sakaguchi, Jun Kurogi, Nana Sakakibara, Kandai Nozu, Hiroshi Moritake

**Affiliations:** ^1^ Department of Pediatrics, Faculty of Medicine, University of Miyazaki, 5200 Kihara, Kiyotake, Miyazaki, 889-1692, Japan, miyazaki-u.ac.jp; ^2^ Department of Pediatrics, Kobe University Graduate School of Medicine, 7-5-1 Kusunoki-cho Chuo-ku, Kobe, 650-0017, Japan, kobe-u.ac.jp

**Keywords:** genetic testing, nephrin, nephrotic syndrome, *NPHS1* variants, steroid-responsive, transient proteinuria

## Abstract

We herein report the case of a 2‐year‐old girl with novel compound heterozygous *NPHS1* variants, p.R460Q (a known loss‐of‐function mutation) and p.V822M (a rare variant with reported pathogenicity and relatively mild functional effects). She initially presented with typical features of idiopathic nephrotic syndrome and achieved complete remission on Day 10 of corticosteroid therapy. After tapering, the patient developed recurrent episodes of infection‐associated heavy proteinuria, which often remitted spontaneously but sometimes left residual low‐grade proteinuria. Despite the introduction of cyclosporine, these episodes continued, and the effect of immunosuppression remained unclear. Over a 4‐year follow‐up, recurrent transient proteinuria persisted, but no relapse of nephrotic syndrome or renal dysfunction was observed. This atypical clinical pattern, characterized by incomplete remissions and limited response to immunosuppressive therapy, prompted genetic testing, which revealed compound heterozygous *NPHS1* variants. This case expands the phenotypic spectrum of *NPHS1*‐associated disease, thus highlighting that nephrin variants may manifest as steroid responsiveness, preserved renal function, and repeated transient proteinuria. Our findings emphasize the role of genetic testing in clarifying diagnosis and guiding management in steroid‐sensitive nephrotic syndrome with atypical features.

## 1. Introduction

Pathogenic biallelic variants in the *NPHS1* gene, encoding nephrin, an essential podocyte protein that maintains the glomerular filtration barrier, typically cause congenital or steroid‐resistant nephrotic syndrome (SRNS) with progressive renal impairment [1]. Nephropathies caused by pathogenic variants are generally steroid resistant, although partial responsiveness has been reported in some cases [2].

In this report, we describe a patient who initially responded to corticosteroids but later developed an atypical course characterized by recurrent episodes of heavy proteinuria that resolved spontaneously. This clinical pattern is not typically observed in idiopathic nephrotic syndrome, thus prompting genetic testing, which revealed compound heterozygous *NPHS1* variants (p.R460Q and p.V822M).

## 2. Case Presentation

A 2‐year‐old girl presented with generalized edema during bronchitis treatment. Urinalysis revealed proteinuria (4+). Her medical history included bronchial asthma, with no family history of kidney disease. Physical examination revealed edema, tachycardia, normal blood pressure, and no dysmorphic features. Laboratory findings included a urinary protein‐to‐creatinine ratio (UP/Cr) of 9.2 g/gCr, serum albumin of 1.1 g/dL, total cholesterol of 460 mg/dL, and preserved renal function (serum creatinine 0.22 mg/dL). The selectivity index was 0.01. The kidney ultrasonography results were normal.

She was diagnosed with idiopathic nephrotic syndrome and treated with prednisolone according to the ISKDC guidelines, achieving complete remission on Day 10. After tapering the prednisolone dose, she experienced frequent episodes of infection‐associated transient heavy proteinuria, which partially improved spontaneously but did not achieve complete remission without additional treatment. She was readmitted, placed in infection‐preventive isolation, and reinduced with high‐dose prednisolone, thereby achieving complete remission.

**FIGURE 1:**
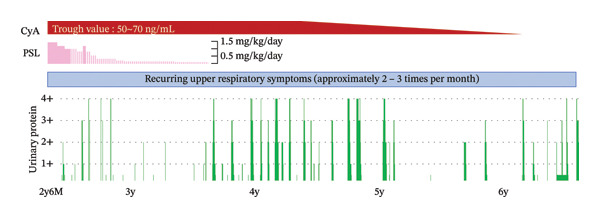
Clinical course of proteinuria and immunosuppressive therapy after discharge. Transient proteinuria recurred during the episodes of infection, while prednisolone and cyclosporine were gradually tapered and eventually discontinued, with no significant change in the clinical pattern. Abbreviations: CyA, cyclosporin A; PSL, prednisolone.

After discharge and during prednisolone tapering, frequent recurrences of transient heavy proteinuria occurred, sometimes achieving complete remission but at other times leaving residual low‐grade proteinuria. This clinical course resembled either persistent proteinuria or steroid‐dependent nephrotic syndrome. Given the clear steroid responsiveness, minimal change nephrotic syndrome was considered most likely; therefore, cyclosporine was introduced. Nevertheless, recurrent episodes continued, and all resolved spontaneously without marked hypoalbuminemia. Cyclosporine was discontinued 2 years later. During the 4 years of follow‐up, the patient continued to show recurrent transient proteinuria, but there was no recurrence of typical nephrotic syndrome or renal dysfunction (Figure 1).

**FIGURE 2:**
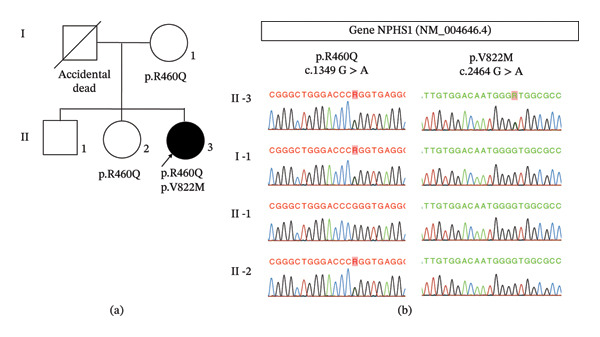
The analysis of the *NPHS1* variant in the family. The patient (indicated by an arrow) carried compound heterozygous *NPHS1* variants, p.R460Q and p.V822M. Her mother and sister carried the p.R460Q variant, while her brother carried neither variant.

Because this course was atypical for idiopathic nephrotic syndrome and the effect of immunosuppressive therapy was unclear, we suspected an underlying genetic cause and thus performed genetic testing. Targeted next‐generation sequencing using a gene panel for proteinuria‐associated disorders was performed, followed by Sanger sequencing for validation of *NPHS1* variants. No pathogenic variants were identified in genes associated with benign or tubular proteinuria, including CUBN. It revealed compound heterozygous *NPHS1* variants c.1379G > A (p.R460Q) and c.2467G > A (p.V822M). Her mother and sister carried p.R460Q alone and were asymptomatic, confirming the presence of compound heterozygosity in the patient (Figure 2).

## 3. Discussion

This case expands the phenotypic spectrum of *NPHS1*‐associated disease. While *NPHS1* variants usually cause congenital nephrotic syndrome or SRNS with progressive renal failure [1, 3], our patient exhibited initial steroid responsiveness, preserved renal function, and frequent infection‐triggered transient proteinuria.

The p.V822M variant has been reported once in sibling cases of nephrotic syndrome with preserved renal function in combination with another *NPHS1* mutation, and the recurrent transient proteinuria observed in that case parallels our patient’s course [4, 5]. In contrast, heterozygous carriers of the p.V822M variant alone did not exhibit proteinuria. The p.V822M variant shows mild functional changes in vitro, whereas p.R460Q has been reported in vivo as a pathogenic mutation causing congenital nephrotic syndrome [3]. Taken together, these findings raise the possibility that p.V822M may have a relatively mild functional impact and contribute to an atypical phenotype, in which steroid responsiveness may be observed despite underlying *NPHS1*‐associated disease. Interestingly, our patient initially exhibited a steroid‐sensitive clinical course. Genome‐wide association studies have identified *NPHS1* as a susceptibility locus for steroid‐sensitive nephrotic syndrome (SSNS), suggesting that genetic variation in the *NPHS1* locus may contribute to disease susceptibility [6]. Although proteinuria associated with genetic abnormalities is generally considered to follow a steroid‐resistant course, our patient, harboring rare *NPHS1* variants, initially exhibited a steroid‐sensitive clinical course. These findings support the concept that *NPHS1*‐associated disease is not limited to severe forms such as congenital nephrotic syndrome or SRNS with renal dysfunction but may encompass a broader phenotypic spectrum, including relatively mild and partially steroid‐responsive presentations.

Paternal genetic analysis was not available; therefore, we cannot exclude the possibility that the p.V822M variant arose de novo.

Our findings indicate that genetic testing in steroid‐responsive cases with atypical features, such as recurrent episodes of spontaneously remitting heavy proteinuria, can refine the diagnosis and guide treatment, as well as help in determining the most appropriate long‐term follow‐up strategies while avoiding unnecessary prolonged immunosuppression. Further accumulation of similar cases is warranted.

## Funding

This work was supported by a Clinical Research Support Grant from University of Miyazaki Hospital.

## Disclosure

This case was presented in abstract form at the 58th Annual Meeting of the Japanese Society for Pediatric Nephrology (2023) and at the 2023 meeting of the Japanese study group for developmental nephrology (Hattatsu‐Jin Kenkyukai).

## Consent

Informed consent for publication was obtained from the patient’s guardian.

## Conflicts of Interest

The authors declare no conflicts of interest.

## Data Availability

The data supporting the findings of this case report are available from the corresponding author upon reasonable request. The data are not publicly available due to privacy or ethical restrictions.
